# Goal-Directed-Behavior in 22q11.2 Deletion Syndrome: Implication for Social Dysfunctions and the Emergence of Negative Symptoms

**DOI:** 10.3389/fpsyt.2020.00230

**Published:** 2020-04-01

**Authors:** Lydia Dubourg, Johanna Maeder, Virginie Pouillard, Stephan Eliez, Maude Schneider

**Affiliations:** ^1^Developmental Imaging and Psychopathology Lab, Department of Psychiatry, School of Medicine, University of Geneva, Geneva, Switzerland; ^2^Department of Genetic Medicine and Development, School of Medicine, University of Geneva, Geneva, Switzerland; ^3^Center for Contextual Psychiatry, Department of Neurosciences, Research Group Psychiatry, KU, Leuven, Leuven, Belgium

**Keywords:** goal-directed-behavior, social functioning, negative symptoms, Cathechol-O-Methytransferase (COMT), 22q11.2 deletion syndrome

## Abstract

**Background:**

Negative symptoms and social dysfunction are core features of the 22q11.2 deletion syndrome (22q11DS). Negative symptoms have been conceptualized as pathology of goal-directed-behaviors. Moreover, goal-directed-behaviors also appear to be a crucial step of social interactions. However, in 22q11DS, the extent to which goal-directed-behavior could be linked to social functioning difficulties and negative symptoms has never been examined.

**Method:**

Verbal and nonverbal initiation was measured using the verbal fluency and figural fluency tasks in 93 individuals with 22q11DS and 57 healthy controls aged between 8 and 30 years in order to assess goal-directed-behavior ability. The associations between initiation scores and social functioning/negative symptoms were investigated. In addition, the effect of COMT Val/Met polymorphism on initiation competences was examined.

**Results:**

Results revealed diminished verbal and nonverbal initiation ability in 22q11DS individuals compared to controls. A positive correlation between verbal initiation and social functioning was found as well as between verbal initiation and negative symptoms, in particular social anhedonia. No differences in terms of initiation scores were found between individuals with 22q11DS carrying Met and Val polymorphism.

**Conclusion:**

Results indicate impaired goal-directed-behavior in the 22q11DS population. These deficits seem to support social functioning impairments frequently observed in the 22q11DS and to a lesser extent the expression of negative symptoms.

## Introduction

Negative symptoms constitute a hallmark of the 22q11.2 deletion syndrome (22q11DS), one of the most frequent microdeletion encountered in a human being (1, 2). As in schizophrenia, research predominantly focused on positive symptoms. Henceforth, positive symptoms can be greatly reduced with medication, while negative symptoms appear to be more persistent and more difficult to treat (3, 4). Given their association with poor socio-occupational functioning (5–7) as their role in predicting transition to psychosis (8, 9), a new interest in understanding the mechanisms underlying negative symptoms emerged.

Negative symptoms encompass a motivational dimension, referring to avolition, anhedonia, and asociality as an expressive dimension consisting of diminished affect and alogia (10). As the motivational dimension is thought to be a more severe aspect of psychopathology (11), numerous studies examined the components of motivation along with their association with negative symptoms [see for a review (12)].

According to Kring and Bach (12), motivation encompasses two distinct key components: value computation (computing the value of a reward) and effort computation (computing how much effort it will take to get the reward); both support a third process called goal-directed-behavior (the actions required to achieve a goal) (12). Up to now, motivational impairments reported in schizophrenia have been argued to reflect issues in translating computations of value and effort into goal-directed-behavior (12). Goal-directed-behavior has thus mainly been studied in the context of the motivational approach, and the ability to initiate actions *per se* has rarely been examined. Only one study has investigated the ability to initiate actions in schizophrenia (13). Through a verbal fluency task, authors demonstrated that individuals with higher negative symptom severity tended to have more pronounced initiation impairments (13).

Yet, some authors proposed that schizophrenia could be considered as a pathology of action (14, 15). In particular, Frith proposed that impaired initiation of willed actions could explain the negative symptoms of schizophrenia (*e.g.*, apathy, anhedonia) (14, 16) Negative symptoms have later been conceptualized as a pathology of goal-directed-behaviors (17).

As most of our actions are directed toward specific goals, it is likely that difficulties in goal setting and achievement might lead to a variety of negative symptoms such as amotivation/apathy and social withdrawal. In addition, goal-directed-behaviors appear to be a crucial step of social interactions (18). Impaired goal-directed-behaviors may also lead to social dysfunction, an additional core aspect of the 22q11DS endophenotype (19, 20). However, the putative association between goal-directed-behavior and negative symptoms or/and social functioning has never been examined in this population.

Moreover, the ability to initiate goal-directed-behavior belongs to a broader set of higher order skills called executive function. In 22q11DS, executive impairments have been extensively demonstrated (21–27). Nevertheless, only one study has examined the ability to initiate actions in 22q11DS (28). Maeder et al. (28) highlighted a different trajectory of verbal initiation with age in individuals with 22q11DS compared to healthy controls as well as a different trajectory of verbal inhibition in participants with negative symptoms (28). Moreover, some genes within the 22q11.2 locus, such as the Catechol-O-Methyltransferase (COMT) gene, are involved in prefrontal functioning, which sustains executive function (29). Thus, haploinsufficiency of some genes could explain the divergent EF competences observed in 22q11DS.

This study aimed to investigate the putative contribution of initiation impairments on social functioning deficits and negative symptom severity in 22q11DS. First, we examined performance on verbal and nonverbal fluency tasks to fit the concept of goal-directed-behavior in 22q11DS and healthy individuals. Indeed, fluency tasks have been well demonstrated to reflect executive control ability (30), which is defined as the set of functions directing behavior toward goals. For this reason fluency tasks can be considered as a proxy for goal-directed behaviors. We expected that 22q11DS individuals would show impaired verbal and nonverbal initiation compared to controls. Based on previous findings, we also hypothesized that initiation deficits would be associated with social dysfunction and negative symptom severity. Secondly, the influence of COMT Val/Met polymorphism on initiation competence was examined. Met polymorphism being associated with accumulation of dopamine, we hypothesized that Met carriers will have lower initiation scores compared to Val carriers.

## Methods

Ninety-tree participants with 22q11DS and 77 healthy individuals aged between 8 and 30 years were included in the study. Healthy controls were screened for the presence of any neurological problems, psychological or learning difficulties, and medication that could influence their performance prior to their inclusion in the study. Some patients met formal diagnostic criteria for a psychiatric condition or were receiving medication at the time of the evaluation (see **Table 1**).

**Table 1:** Demographic data of the participants.

		Diagnostic group		Comparison	
		22q11DS	Controls	Anova	p-value
N		93	77		
Age		17.4 (6.1)	15.3 (5.5)	5.07	0.026
Gender (% of female)		49.5	51.9	0.104	0.747
Full IQ [mean (SD)] SIPS negative SIPS positive Verbal fluency total score Figural fluency		72.1 (13.6)	113 (13)	407.4	<0.001
		2.25 (0.86) 1.46 (0.83) 9.5 (3.2) 23.2 (9.3)	/ / 12.7 (4.6) 33 (10.2)	28.6 42.9	<0.001 <0.001 <0.001
SRS [mean (SD)]	SRS awareness	62 (12.6)	49.9 (11.3)	37.7	<0.001
	SRS cognition	65.2 (13)	45.4 (6.9)	129.7	<0.001
	SRS communication	62.2 (13.3)	45.9 (6.8)	85.4	<0.001
	SRS motivation	61.2 (12.7)	47.2 (7.5)	65.8	<0.001
	SRS RRB	64 (13.9)	43.8 (6.7)	123.3	<0.001
	SRS Total	66 (17.5)	45.7 (6.5)	82.7	<0.001
Psychiatric diagnosis [N (%)]	Specific phobia	19 (20%)			
	ADHD	29 (31%)			
	Generalized anxiety disorder	14 (15%)			
	Major depression disorder	12 (13%)			
	Social phobia	2 (2%)			
	Obsessive compulsive disorder	2 (2%)			
	Schizophrenia	5 (5%)			
	Schizoaffective disorder	2 (2%)			
	Dysthymic disorder	1 (1%)			
	Panic disorder	1 (1%)			
	Separation anxiety disorder	2 (2%)			
Psychotropic medication	Antipsychotics	16 (17%)	0		
	Antidepressants	18 (19%)	0		
	Psychostimulants	14 (15%)	0		

SIPS, Structured Interview for Prodromal Syndromes; SD, Standard Deviation; SRS, Social Responsiveness Scale; ADHD, Attention Deficit Hyperactivity Disorder.

Patients were recruited through French-speaking parent associations and were tested during an ongoing longitudinal study. Written informed consent was obtained from participants and their parents under protocols approved by the Geneva cantonal ethics commission of research.

The presence of psychiatric disorders was evaluated using the Diagnostic Interview for Children and Adolescent—Revised and the mood and psychosis supplement of the Kiddie-Schedule for Affective Disorders and Schizophrenia Present and Lifetime version [K-SADS-PL (31)] in adolescents below 18 years and the Structured Clinical Interview for DSM-IV axis disorders [SCID-I (32)] for adults. The presence of positive and negative symptoms of schizophrenia was assessed using the Structured Interview for Prodromal Syndromes [SIPS (33)]. Based on a previous study exploring the factor structure of the SIPS in 22q11DS (34), only the SIPS scores of the item loading on the negative factor (N1 to N4 and D4) were used in this study.

Polymorphism of the COMT gene was determined by polymerase chain reaction restriction fragment length polymorphism analysis for 63 participants with 22q11DS (35). Thirty-five participants were Met 158 homozygous and 28 were Val 108. The two groups did not differ in age [F (1,62) = 3.803, p= 0.056] or gender distribution [X^2^ (1,57) = 0.204, p= 0.651].

Full-scale IQ was measured for all participants using the Wechsler Intelligence Scale for children 3^rd^ or 4^th^ edition [WISC-III-R (36) or IV (37)], or the Wechsler adult Intelligence Scale 3^rd^ or 4^th^ edition [WAIS-III (38) or IV (39)] to obtain an evaluation of global intellectual functioning.

Verbal initiation was assessed using a verbal fluency task (40, 41). The version encompasses a phonemic fluency task (using the letters FAS) and a semantic part (animal and food category). We used the number of words produced, which reflects the efficiency in initiating a verbal content. A total score including all subcategories and representing a global measure of verbal initiation was calculated. The number of repetitions and rule violations of all subcategories was also calculated and used for statistical analyses. To assess a nonverbal counterpart to verbal initiation, we used an adaptation of the 5-point task (42, 43). Participants were asked to draw as many drawings as possible on a 5-point structure during 3 min, without repeating the same drawing twice. We used the total number of different drawings (as a measure of nonverbal initiation), repetitions, and rule violation scores for statistical analyses.

Social functioning was assessed by administrating the 2^nd^ edition of the Social Responsiveness Scale [SRS-2; (44)] to parents of 87 participants with 22q11DS and 69 controls to identify the presence and severity of social impairments. Data were missing for 14 individuals. The SRS-2 includes five domains: social awareness, social cognition, social communication, social motivation, and restricted interests as well as repetitive behavior. Raw scores for each scale are converted to a gender-specific T score representing the individual’s social behavior impairment. The five scales are summed and converted into a T score, resulting in an overall composite score. Higher scores suggested greater social impairment.

All analyses were conducted in SPSS version 22 (IBM Corp., USA). Differences in terms of initiation were examined using ANCOVAs with age as covariate, given its significant differences between groups (F = 5.07, p = 0.026). We examined in each group how initiation performances are associated with age by conducting partial correlation with full-scale IQ as covariate. Moreover, in view of our large age range and to better understand the effect of age distribution on our results, our sample size was divided according to the median split of our sample (median split = 17). Thus, 43 individuals with 22q11DS and 45 healthy controls were included in the group below 16 years old, and 50 individuals with 22q11DS and 32 healthy controls were included in the group above 16 years old. As both groups did not differ in terms of age and gender distribution (all p < 0.05), we used ANOVAs to compare verbal and nonverbal initiation scores between controls and 22q11DS individuals in each age group. Then, we compared the strength of the correlation between groups using Fisher r-to-z-transformation. To ensure that the differences in verbal and nonverbal initiation between 22q11DS individuals and healthy controls truly reflected impairment in goal-directed behavior and were not related to potential confounds, we examined the influence of processing speed, using the processing speed index of the Wechsler scale, the visuoconstructive ability, using the block design subtest of the Wechsler scale, as well as the influence of working memory, using the working memory subtest of the Wechsler scale on both verbal and nonverbal initiation tasks. The influence of lexical knowledge and lexical use, using the vocabulary subtest of the Wechsler scale, on verbal initiation performance was also examined. First, we compared differences in processing speed, block design, working memory, and vocabulary scores between groups using ANCOVA with age as covariate. We then conducted once again the analyses (ANCOVAs) with processing speed index (for both verbal and nonverbal initiation), block design (for nonverbal initiation only), working memory (for both verbal and nonverbal initiation), and the vocabulary index (for verbal initiation only) as covariates.

To test the influence of the COMT polymorphism on initiation scores, ANOVAs comparing verbal and nonverbal initiation scores between Met and Val carriers were conducted. The impact of initiation on social functioning in 22q11DS was investigated by conducting hierarchical stepwise regression analyses. To examine the relationship between negative symptoms (SIPS subscales) and initiation scores in 22q11DS, we conducted partial correlations with full-scale IQ, age, and gender as covariates.

## Results

### Initiation Competences

**Figure 1:**
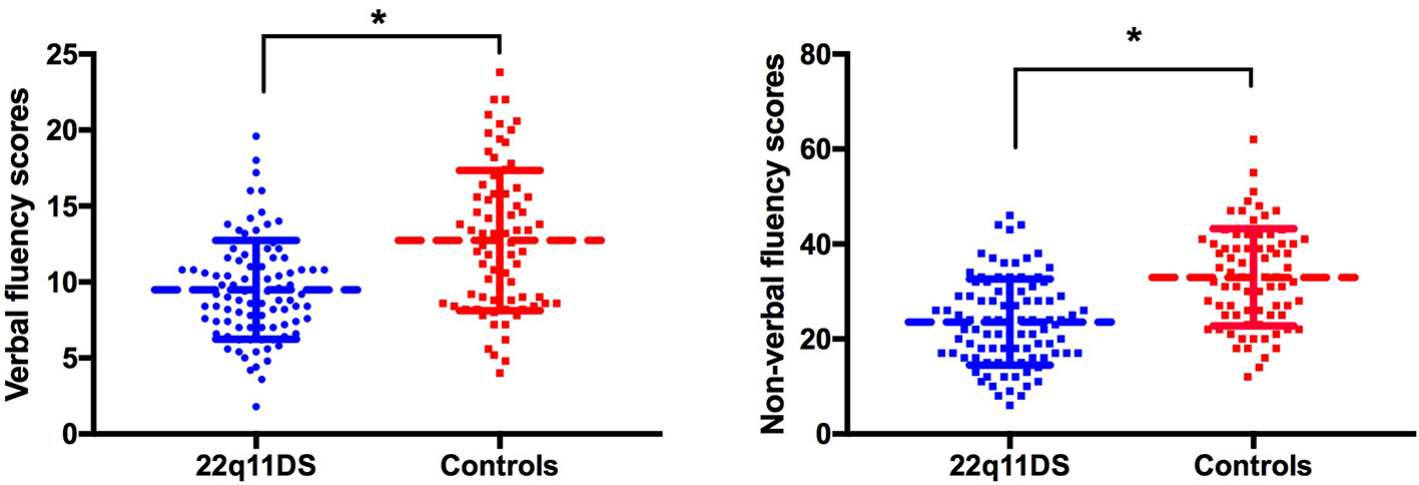
Group comparison of verbal (left) and nonverbal initiation (right). Raw scores are displayed on the vertical axis. *p < 0.05.

Group comparisons (**Figure 1**) revealed that patients with 22q11DS had significantly lower verbal (F_(1,167)_ = 61.8, p < 0.001) and nonverbal (F_(1,167)_ = 65.5, p < 0.001) initiation performance compared to healthy controls. Regarding repetitions, results revealed that 22q11DS individuals and healthy controls produced similar number of repetitions for both verbal [F (1,164) = 0.033, p = 0.855] and nonverbal initiation tasks [F (1, 149) = 1.84, p= 0.177]. Patients with 22q11DS made equivalent number of rule violations for the verbal initiation task [F (1,164) = 0.64, p= 0.426] compared to healthy controls, while for the nonverbal initiation task a higher number of rule violations were observed in the 22q11DS group [F (1,148) = 4.54, p = 0.035].

### Association Between Age and Initiation Performances

By investigating how initiation performance is associated with age, we observed strong associations between age and verbal initiation (r = 0.620, p < 0.001) as nonverbal initiation (r = 0.834, p < 0.007) in healthy controls. In individuals with 22q11DS, significant associations between age and verbal initiation (r = 0.400, p < 0.001) and nonverbal initiation (r = 0.278, p = 0.007) were also found. Nevertheless, comparison of the strength of the correlation between groups revealed that individuals with 22q11DS exhibited weaker association between age and verbal and nonverbal initiation compared to healthy controls (respectively, z = 1.92, p = 0.027 and z = 5.83, p < 0.001). When comparing initiation performances in the group below 16 years old, results revealed that 22q11DS individuals had significantly lower verbal [F (1,85) = 14.14, p < 0.001] and nonverbal [F (1,85) =21.46, p < 0.001] initiation performance. In the group above 16 years old similar results were found {verbal initiation [F (1,79) = 63.76, p < 0.001], nonverbal initiation [F (1,79) = 56.64, p < 0.001]}.

### Relationship Between Initiation Performances and Potential Confounds

Comparison of processing speed, block design, digit span, and vocabulary scores index between groups revealed that participants with 22q11DS exhibit lower scores on the processing speed [F (1, 161) = 87.33, p < 0.001], block design [F (1, 168) = 224.19, p < 0.001], digit span [F (1, 168) = 47.85, p < 0.001] and vocabulary subtests [F (1, 158) = 213.88, p < 0.001] compared to healthy controls.

We compared once again group differences in terms of initiation performances by taking into account differences in processing speed, visuoconstructive ability (*i.e.* block design subtest), working memory (*i.e.* digit span subtest), and lexical knowledge and use (*i.e.* vocabulary subtest). Results demonstrated that performance in verbal [F (1,164) = 24.95, p < 0.001] and nonverbal initiation [F (1,152) = 8.71, p = 0.004] remained significantly different between groups.

### Influence of COMT Polymorphism on Initiation Competences

Results revealed that individuals with 22q11DS carrying Met and Val polymorphism did not differ in terms of verbal [F (1,62) = 0.318, p = 0.575) and nonverbal initiation scores [F (1,62) = 0.182, p = 0.671].

### Association Between Initiation and Social Functioning

In individuals with 22q11DS, hierarchical regressions revealed a significant association between verbal initiation scores and the SRS-2 total, SRS-2 communication, SRS-2 awareness, and the SRS-2 cognition subscales (**Table 2**). No significant association between nonverbal initiation and the SRS-2 subdomains was observed. After Benjamini–Hochberg correction, only the association between verbal initiation and the SRS-2 awareness, SRS-2 communication, and SRS-2 cognition remained significant.

**Table 2:** Hierarchical regression models examining the association verbal and non-verbal initiation and social functioning in patients with 22q11DS.

Dependent variables	Model		Coefficients				
Significant independent variable	R	F (1,57)	b	SE b	*β*	t	p
**SRS-2 total** Nonverbal initiation Verbal initiation	0.385	7.56	−0.122 −1.423	0.211 0.636	−0.63 −0.257	−0.577 −2.236	0.565 0.028*
**SRS-2 motivation** Nonverbal initiation Verbal initiation	0.392	0.153	−0.221 −0.698	0.158 0.478	−0.159 −0.174	−1.395 −1.461	0.167 0.148
**SRS -2communication** Nonverbal initiation Verbal initiation	0.372	0.138	0.008 −1.361	0.168 0.506	0.005 −0.324	0.047 −2.693	0.963 0.009**
**SRS-2 awareness** Nonverbal initiation Verbal initiation	0.333	3.44	0.224 −1.517	0.161 0.487	0.162 −0.381	1.389 −3.114	0.169 0.003**
**SRS-2 cognition** Nonverbal initiation Verbal initiation	0.420	0.176	0.133 −1.345	0.161 0.487	0.092 −0.326	0.825 −2.764	0.412 0.007**
**SRS-2 restricted interest** Nonverbal initiation Verbal initiation	0.299	0.089	−0.111 −0.491	0.181 0.544	−0.072 −0.112	−0.615 −0.902	0.540 0.370

Model with full-scale IQ score as predictors. *p < 0.05, **significant after Benjamini–Hochberg correction.

### Influence of Initiation Performance on Negative Symptoms

Information about negative symptoms (SIPS) was available for 81 participants.

By examining the putative association between initiation scores and negative items, we found a significant negative correlation between negative total score and verbal initiation scores in participants with 22q11DS (r = −0.228, p = 0.044). Particularly, a strong negative association between verbal initiation and N1 ‘social anhedonia’ was observed (r = −0.307, p = 0.007). Regarding nonverbal initiation, none of the tested correlation reached a significant threshold (all p > 0.05).

## Discussion

This study aimed to bring out the contribution of initiation impairments on social dysfunction and negative symptoms in 22q11DS. Findings revealed deficits in both verbal and nonverbal initiation in 22q11DS compared to healthy individuals. Initiation impairments were predictive of social functioning level in 22q11DS individuals. Moreover, verbal initiation impairment was associated with the severity of negative symptoms in 22q11DS individuals.

Our results confirmed deficits in initiation processes in 22q11DS. These results are in agreement with previous studies demonstrating divergent verbal initiation in individuals with 22q11DS compared to healthy controls (28). For the first time, nonverbal initiation was investigated in 22q11DS and was demonstrated as impaired. As mentioned previously, initiation competences belong to a broader set of processes called executive function. In 22q11DS, the variability in EF has been linked to allelic variation in genes related to dopamine metabolism and regulation (45). For this reason, we tested whether the Met/Val polymorphism of the Cathechol-O-Methyltransferase (COMT), a gene critically involved in the dopamine pathway and located in the 22q11.2,may explain divergent EF competences observed in 22q11DS. Contrary to our expectation, we observed that patients with Met and Val polymorphism did not differ in terms of verbal and nonverbal initiation competences. Nevertheless, in view of the small sample size of the COMT genotype subgroups, further studies examining this association are required. Moreover, EF capacities mainly rely on the prefrontal cortex (PFC) (46). In healthy individuals, refinement of the PFC (synaptic pruning and/or myelination) is thought to be related to increase EF competencies in early adulthood (47). In 22q11DS, a delayed prefrontal maturation has been shown and may explain the observed EF impairments (48–50).

Our results support the hypothesis that impaired ability to initiate actions contributes to the emergence of clinical manifestations in 22q11DS. Indeed, we reported that verbal initiation impairment particularly predicts lower social functioning in 22q11DS individuals. In other population marked by social deficits, such as Autism Spectrum Disorders (ASD) or schizophrenia (SZ), the link between impaired executive function and social deficits has been clearly identified. Moreover, the role of initiation in social deficits has been particularly emphasized (51–53). Indeed, significant associations between impaired initiation and reduced social functioning and skills (51, 52, 54, 55) have been observed in individuals with ASD. A strong association between verbal fluency and social competence has also been observed in SZ (53). To our knowledge, the present study is the first to clearly distinguish between verbal and nonverbal initiation. Indeed, in previous studies on ASD, initiation scores extracted from a parent-reported questionnaire included items evaluating both verbal and nonverbal initiation. In SZ, only verbal initiation and its association with social functioning have been examined. Thus, the contribution of each component separately on social functioning remains unclear.

Finally, our findings are also in line with our hypothesis suggesting that initiation could represent a key aspect in negative symptoms’ emergence. Indeed, a significant correlation between verbal initiation and negative symptoms, especially with social anhedonia, has been observed in 22q11DS participants. This finding is coherent with previous work showing a link between some EF domains, including verbal initiation, and negative symptoms in 22q11DS (28). The authors found that compared to individuals without negative symptoms, those with negative symptoms did not exhibit an improvement of verbal initiation with age (28). This result suggests that initiation impairment could precede the onset of negative symptoms. Taken together, the current results and previous work support the conceptualization of negative symptoms as a ‘pathology of goal-directed-behavior’ and suggest that initiation processes might underlie the emergence of negative symptoms in 22q11DS. As negative symptoms represent one of the main consequence of poor daily-life functioning and vocational outcome in this population (56), intervention techniques targeting initiation processes should be considered. Nevertheless, given the preliminary nature of this study, the results should be interpreted carefully and future studies examining the association between goal-directed behavior and negative symptoms in the field of psychosis are required.

There are several limitations in this study. First, we used fluency tasks, which reflect executive control ability, as a proxy of goal-directed-behavior. Nevertheless, additional processes such as decision making and cognitive control, which cannot be accessed through fluency tasks, may play a considerable role in goal-directed-behavior achievement. Thus, further studies examining the implication of decision making and cognitive control should be conducted. Secondly, we did not observe a significant association between N2 ‘Avolition’ and initiation performances in our results, However, as the avolition domain of negative symptoms has high validity with initiation, this raises a question regarding the use of fluency tasks as proxy of goal-directed-behavior. Further studies using more ecological tasks are therefore required to confirm those findings. Thirdly, some patients where under medication at the time of the testing. Some drugs having an influence on the dopamine system, the effect on the current results should be investigated. Nevertheless, in view of the variability of molecules and dosage across participants, it was not possible to examine the effect of types of medication in this study.

Fourthly, individuals with 22q11DS were marked by high rates of comorbid symptoms, which might impact our results. Indeed, in ADHD individuals lower scores in verbal fluency have been previously observed compared to typical developing controls [*e.g.* (57)]. It is also likely that anxiety or depressive symptoms have an impact on the motivation of patients to engage in goal-directed behavior. Nevertheless, as a majority of patients with 22q11DS presented several comorbid symptoms simultaneously or through lifespan, it was impossible to study the effect of comorbidity in this study.

Finally, although autism appears to be very common in this population (58), we did not perform a formal diagnosis of autism in this study and could therefore not examine the impact of autism comorbidity on our results.

The current study pointed out impairment in verbal and nonverbal initiation in the 22q11DS population. It appears that initiation deficits are associated with social dysfunction of 22q11DS individuals. Findings also suggest that initiation impairment is associated with negative symptoms emergence severity in this population.

## Data Availability Statement

The raw data supporting the conclusions of this article will be made available by the authors, without undue reservation, to any qualified researcher.

## Ethics Statement

This study was carried out in accordance with the recommendations of ’name of guidelines, name of committee’ with written informed consent from all subjects. All subjects gave written informed consent in accordance with the Declaration of Helsinki. The protocol was approved by the cantonal ethics commission of research.

## Author Contributions

LD and JM designed the study. LD, JM, VP, MS, and SE administered the tools. LD carried out the data analysis and wrote the manuscript. All authors read and approved the final manuscript.

## Funding

This work was supported by research grants from the Swiss National Science Foundation (grant no. 324730_121996) for SE, (grant no. PZ00P1_174206) for MS, and The National Center of Competence in Research “Synapsy - The Synaptic Bases of Mental Diseases” to SE (grant no. 51NF40-185897).

## Conflict of Interest

The authors declare that the research was conducted in the absence of any commercial or financial relationships that could be construed as a potential conflict of interest.
